# Efficacy of combined targeted radionuclide therapy and immune checkpoint Inhibition in animal tumour models: a systematic review and meta-analysis of the literature

**DOI:** 10.1007/s00259-025-07293-0

**Published:** 2025-04-26

**Authors:** Simone C. Kleinendorst, Carlijn R. Hooijmans, Stijn Muselaers, Egbert Oosterwijk, Mark Konijnenberg, Sandra Heskamp, Sanne A. M. van Lith

**Affiliations:** 1 Department of Medical Imaging, Nuclear Medicine, Radboud University Medical Center, Nijmegen, The Netherlands; 2 Department of Anesthesiology, Pain and Palliative Medicine, Radboud University Medical Center, Nijmegen, The Netherlands; 3 Department of Urology, Radboud University Medical Center, Nijmegen, The Netherlands; 4 Department of Radiology and Nuclear Medicine, Erasmus MC Cancer Institute, Erasmus University Medical Center, Rotterdam, The Netherlands

**Keywords:** Targeted radionuclide therapy, Immune checkpoint inhibition, Combined modality therapy, Systematic review, Meta-analysis

## Abstract

**Purpose:**

Given radiation’s immunomodulatory effects and the complementary anti-cancer mechanisms of targeted radionuclide therapy (TRT) and immune checkpoint inhibition (ICI), their combination holds promise as a cancer treatment. This systematic review and meta-analysis summarize the literature on the therapeutic efficacy of combined TRT/ICI in animal tumour models.

**Methods:**

A systematic search in MEDLINE-PubMed and Embase-OVID was performed. Study characteristics and risk of bias were assessed. Outcome parameters included normalized area under the tumour growth curve and restricted mean survival time, of which ratios between combined treatment and untreated and monotherapy groups were analysed in a random-effects meta-analyses. Predefined subgroup analyses explored potential moderators of treatment efficacy.

**Results:**

In total, 31 studies were included. Study characteristics such as animal sex and age, cancer type, TRT target, and radionuclides, varied considerably across studies. The quality of the included studies could not always be assessed due to poor reporting. All meta-analyses indicated significantly improved survival and tumour growth of combination treatment over untreated, TRT and ICI monotherapy controls (RMST ratio 1.96 [1.72–2.23], 1.44 [ 1.34–1.55], 1.54 [1.38–1.72], and nAUC ratio 0.32 [0.25–0.42], 0.49 [0.41–0.59], 0.41 [0.31–0.55], respectively), with high between-study heterogeneity (I^2^ = 76.7–98.2%). The specific mode of action of ICI emerged as a potential moderator of treatment efficacy in subgroup analyses.

**Conclusion:**

This systematic review highlights the therapeutic potential of combined TRT/ICI treatment, demonstrating preclinical proof-of-concept and supporting its further evaluation in clinical trials. However, the current literature remains insufficient to determine optimal treatment parameters like TRT tumour-absorbed dose and ICI type for clinical translation. Further research with improved reporting standards should systematically evaluate the impact of such parameters to enable robust comparisons.

**Supplementary Information:**

The online version contains supplementary material available at 10.1007/s00259-025-07293-0.

## Introduction

Targeted radionuclide therapy (TRT) is a systemic cancer treatment in which radioactive atoms are delivered to tumour cells by tumour-targeting agents such as antibodies, peptides, or small molecules [1, 2]. This allows irradiation of all antigen-positive tumour sites at the same time and results in tumour cell death via DNA- and cellular damage. TRT can be applied to various cancer types, including patients with (widespread) metastatic disease. Clinical use of TRT has mainly focused on the treatment of neuroendocrine tumours and prostate cancer with the FDA approval of ^177^Lu-labeled DOTATATE (Lutathera^®^) and, more recently, ^177^Lu-labeled PSMA-617 (Pluvicto^®^), respectively [2]. Additionally, many new TRT agents are being developed to target antigens in the tumour microenvironment (TME). For example, various TRT agents directed to fibroblast activation protein (FAP), which is expressed on cancer-associated fibroblasts (CAFs), have been developed and first clinical studies are being conducted to test its efficacy in cancer patients [3]. Despite TRT’s promising results, durable responses are limited to subgroups of patients. Therefore, there is a clear need to improve TRT efficacy. Consequently, clinical and preclinical studies have started exploring the use of alpha- rather than beta-emitting radionuclides, because alpha particles have a higher cytotoxic potential due to their higher energy and shorter range. Furthermore, numerous combination strategies are currently explored, for example using immunotherapy [4].

Different variants of immunotherapy exist, among which immune checkpoint inhibitors (ICI) are an important class. ICI have been implemented in clinical routine as therapy for various cancer types since the first FDA approval in 2011 [5]. ICI are antibodies which bind to co-inhibitory proteins present on immune or tumour cells, thereby reversing immune suppression and reactivating the patient’s anti-tumour immunity. The majority of FDA-approved antibodies target cytotoxic T-lymphocyte associated protein-4 (CTLA-4, e.g. ipilimumab), programmed cell death-1 (PD-1, e.g. pembrolizumab, nivolumab, cemiplimab, dostarlimab, toripalimab), or programmed death ligand-1 (PD-L1, e.g. atezolizumab, avelumab, durvalumab), and they are often used in combination. Application of ICI treatment includes, but is not limited to, melanoma, non-small cell lung cancer, renal cell carcinoma, and colorectal cancer. However, limited durable response rates and resistance to ICI underscore the importance of combination strategies.

The immunomodulatory potential of radiation has long been recognized, driving clinical studies on combined external beam radiation therapy (EBRT) and ICI treatment such as the phase III PACIFIC trial [6]. With the advantageous application of TRT for metastatic cancer, various exploratory phase I and phase II clinical trials on combined TRT/ICI treatment have been initiated over the past few years. The majority of these studies is focused on combination with Lutathera^®^ (NCT04261855, NCT03457948, NCT04525638, NCT05583708, and [7]) and Pluvicto^®^ (NCT05150236, NCT05766371, and [8, 9]). Furthermore, some trials are assessing combinations of ICI with novel TRT agents such as ^225^Ac-labeled PSMA-targeting antibody J591 (NCT04946370) for metastatic prostate cancer patients and ^177^Lu-labeled CAIX-targeting antibody girentuximab for advanced clear cell renal cell carcinoma patients (NCT05663710, NCT05239533). These ongoing clinical trials typically adhere to established dosing regimens, often using the clinically approved activity of the radiopharmaceutical (e.g. 7.4 GBq for Lutathera^®^ and Pluvicto^®^), and treatment sequencing is largely based on existing clinical protocols. However, the therapeutic efficacy of combined TRT/ICI can be influenced by multiple factors, including the type and tumour-absorbed dose of TRT and ICI, as well as the order and sequence of administration [10]. Preclinical studies are actively investigating these variables, for instance by exploring whether lower activity levels of TRT, which are subtherapeutic as monotherapy, can render tumours more immunogenic and responsive to ICI, potentially reducing toxicity while maintaining efficacy. Additionally, an important area of research is the different sequencing strategies, such as administering TRT before ICI to enhance immune priming and improve treatment outcomes.

A systematic review of these studies is therefore highly timely to evaluate the current evidence and identify key parameters that contribute to treatment efficacy. This will not only provide an important overview of the animal models, cancer types, radionuclides, and targets under investigation, but also offer insights into optimal treatment strategies that could guide future preclinical and clinical trial designs.

In this report, we present a systematic overview and meta-analysis of all available preclinical evidence on the effect of combined TRT/ICI therapy on tumour growth and survival in animal tumour models compared with untreated, TRT-treated, or ICI-treated control animals.

## Methods

This systematic review was registered in the International prospective register of systematic reviews PROSPERO (CRD42023476424), from which the review protocol can be accessed. This systematic review is reported according to the Preferred Reporting Items for Systematic Reviews and Meta-Analyses (PRISMA) statement.

### Literature search strategy and study selection

A comprehensive search strategy for MEDLINE (via PubMed) and EMBASE (via Ovid) was created. Four search components were included: (1) non-human animal species; (2) cancer; (3) targeted radionuclide therapy; (4) immune checkpoint inhibition. Each component included thesaurus terms and free text terms (complete search string in Supplementary material 1.1). For search component 1, previously developed PubMed and EMBASE search filters were used [11]. For EMBASE, the search was limited to publication types articles or articles in press, with no language restrictions applied.

The search was conducted on November 1st 2023 and updated on July 1st 2024. Duplicates were removed. The search results were screened in two phases, title/abstract and full-text screening, independently by two reviewers (SCK and SvL) using Rayyan [12]. Disagreements in classification between the two reviewers were discussed until consensus was reached. No further publications were included following screening of reference lists of included studies and relevant reviews.

### Inclusion and exclusion criteria

Specific study inclusion and exclusion criteria are available from Supplementary material 1.2. In short, inclusion criteria were: (1) original controlled experimental animal studies, (2) experimental animals with tumours, (3) combined targeted radionuclide therapy and immune checkpoint inhibition treatment, and (4) tumour growth data and/or survival data.

In the title/abstract screening phase, exclusion criteria were: (1) no original research, (2) no animal model, (3) no tumour model, (4) no or other type of radiotherapy, (5) no or other type of immunotherapy. In the full-text screening phase, studies were excluded for the same criteria, and additionally for (6) no combination therapy, (7) no control group, (8) neither tumour growth nor survival data.

### Study characteristics

Information and characteristics from all included studies were extracted, including the animal model (e.g. species, sex, age, tumour induction method, cancer type), the intervention (e.g. target, radionuclide, administration route, activity, tumour-absorbed dose, type of ICI, treatment schedule), and outcome characteristics (e.g. type of outcome, tumour size measurement method, and follow-up time). Graphs were created using GraphPad Prism, version 5.03.

### Risk of bias analysis

Methodological quality of the included studies was assessed using an adapted version of SYRCLE’s risk of bias tool for animal studies [13]. Reporting questions on randomization, blinding, sample size calculation and conflict of interest were added to the items of the bias tool. The assessment focused exclusively on the in vivo combination therapy experiments. Risk of bias analysis was performed independently by two reviewers (SCK and SvL). Disagreements between the two reviewers were discussed until consensus was reached.

### Data extraction

Two outcome measures were used for statistical analysis; survival and tumour growth. All authors were contacted by e-mail to provide raw data, if not yet available. If the requested data were not obtained, data were extracted manually from the graphical representations using ImageJ (1.53t, National Institutes of Health, USA). If the graphs were not sufficient to extract data manually and raw data was not available, comparisons were excluded from the meta-analysis. After data extraction (SCK), included data were peer-reviewed (SvL). In order to assess survival, the number of events per timepoint was extracted. For tumour growth, tumour volumes for each reported timepoint were extracted (mean, SEM or SD, n). If possible, the sample size (n) per timepoint was determined using the survival data. Further assumptions during data extraction are described in Supplementary material 1.3.

### Data analysis

For detailed reasoning for the choice of used analysis parameters, see Supplementary material 1.4. In order to analyse tumour growth, the original graphs were reproduced in Graphpad Prism 9, and mean area under the curve (AUC) for each group was determined. The AUC was normalised for the study duration to take into account the follow-up time that varied across groups and studies (nAUC).

The nAUC ratio (treatment versus control) and its SEM were calculated by using the following formula:

$$\:SE{M^{ratio}} = \:\frac{{nAU{C_{exp}}}}{{nAU{C_{ctrl}}}} \cdot \:\sqrt {{{\left( {\frac{{S{E_{exp}}}}{{nAU{C_{exp}}}}} \right)}^2} + {{\left( {\frac{{S{E_{ctrl}}}}{{nAU{C_{ctrl}}}}} \right)}^2}} ).$$

An nAUC ratio < 1 indicates lower tumour growth in the experimental group, while a ratio > 1 indicates lower tumour growth in the control group.

For survival analysis, restricted mean survival time (RMST) was calculated using the ‘survRM2’ package in R [14]. RMST represents the average survival time up to a specified time point, which was defined as end of follow-up in each study. The RMST ratio (treatment versus control) and its 95% confidence interval were used to assess treatment efficacy, where a ratio > 1 indicates better survival in the experimental group, while a ratio < 1 indicates a better survival in the control group.

If a study included multiple experimental conditions in different groups of animals, each was separately compared with the control. Data were pooled using a random-effects model and visualized in forest plots using Comprehensive Meta-Analysis Version 4 [15]. Plots were further formatted in Adobe Illustrator. Between-study heterogeneity was assessed using the I^2^ statistic.

Investigations of heterogeneity were performed using mixed-effects subgroup analyses, for predefined modulators with data from at least ten independent studies (Supplementary material 2). To detect the risk of publication bias, funnel plots were visually inspected following Duval and Tweedie trim and fill analysis. To assess the robustness of our findings for tumour growth, a sensitivity analysis was performed for two methods used to calculate the SEM of nAUC:

Method 1: $$\:{SEM}^{nAUC}=\:\frac{{SEM}_{AUC}}{study\:duration}$$

Method 2: $$\:SE{M^{nAUC}} = \:\frac{1}{{\sqrt {n - 2} }} \cdot \:\sqrt {\frac{{\sum \: {{({y_i} - \hat y)}^2}}}{{\sum \: {{({x_i} - \mathop x\limits^ - )}^2}}}} $$

CMA files are provided in Supplementary material 3-11.

## Results

### Adjustments to the protocol

Discrepancies between the two independent reviewers during screening and risk of bias analysis could be resolved by discussing until consensus was reached, therefore, no third reviewer was addressed. The outcome measures for tumour growth and survival were adjusted from standardized mean difference and hazard ratio to nAUC ratio and RMST ratio, respectively, for which extensive reasoning can be found in Supplementary material 1.4.

### Study selection

**Fig. 1:**
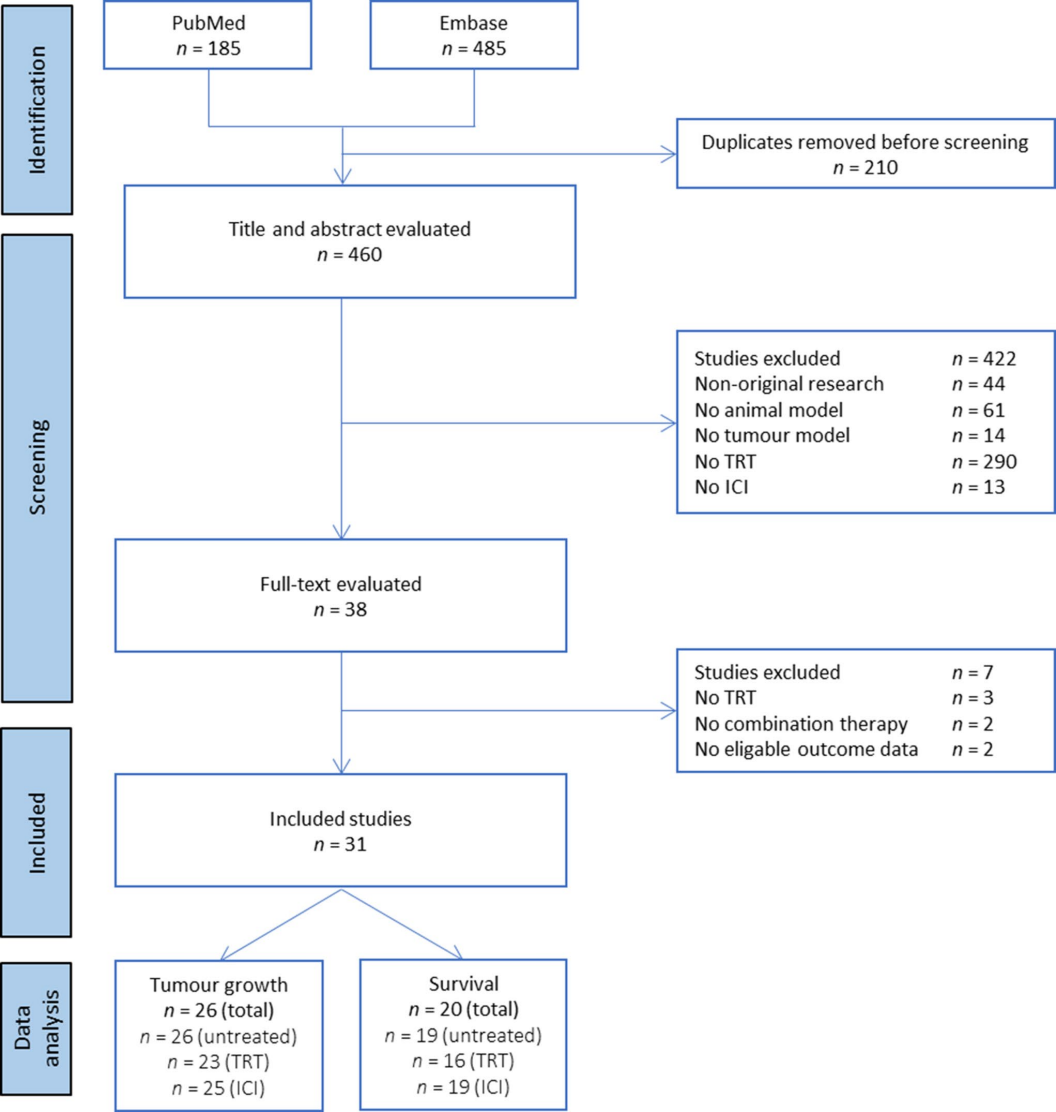
PRISMA flow diagram for study selection

Articles on the efficacy of combined TRT/ICI evaluated in animal tumour models were assembled using search strategies, which resulted in a total of 670 articles, 185 from PubMed and 485 from Embase (Fig. 1). After removal of 210 duplicates, a total of 460 articles were screened for title and abstract, of which 422 were excluded according to predefined exclusion criteria. The remaining 38 articles were screened by full-text and subsequently another seven articles were excluded. Ultimately, this led to a final inclusion of 31 articles [16–46]. In total, 26 studies reporting tumour growth and 20 studies reporting survival were included in the data analysis.

### Study characteristics

**Fig. 2:**
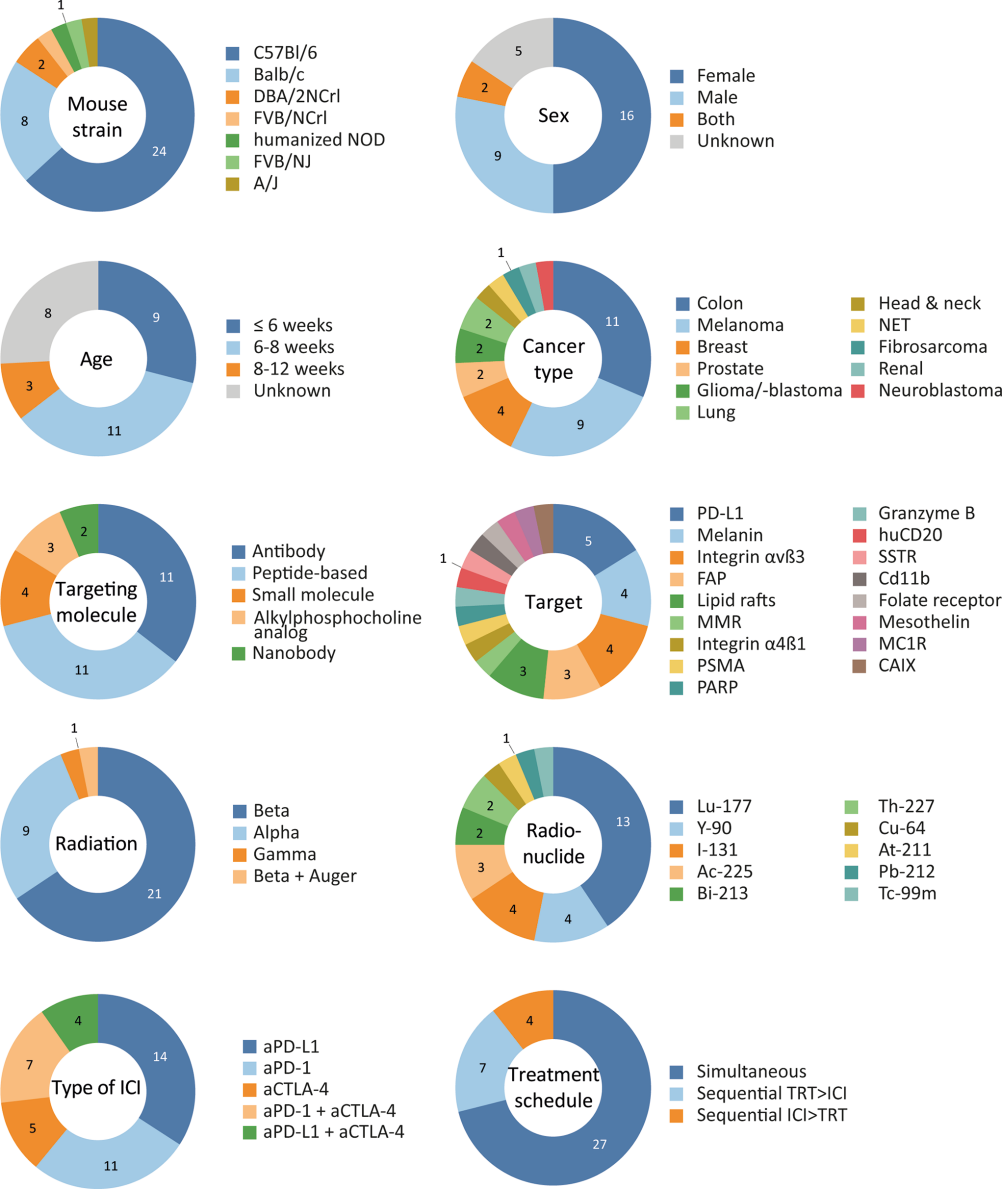
Schematic depiction of a selection of data characteristics, including animal model and intervention characteristics. Numbers indicate the amount of studies investigating the indicated parameter. Of note, some studies investigated multiple parameters, resulting in a total number larger than the total included studies of 31

The characteristics of the animal models and interventions used in the included studies are summarized in the data characteristics table (Supplementary material 12) and Fig. 2.

Although the search included all non-human animal species, all included studies used a mouse model. Tumours were grown in a variety of mouse strains of which C57BL/6 and Balb/c were most frequently used. Sixteen studies were conducted in female mice, while male mice were used in nine studies and mixed sexes were used in two studies. In the other studies, sex was not reported. The reported age of mice was mostly below 6 weeks or between 6 and 8 weeks, although in most cases, it was unclear whether this referred to the time of arrival, the start of experiment, or the beginning of treatment. Moreover, in many studies, age was not reported at all. Twenty-nine studies used subcutaneous tumour models, while one study used an orthotopic and one a metastatic intravenous tumour model. These were all solid tumour models, of which colon adenocarcinoma and melanoma were the most frequently used tumour types. Other investigated tumour types included breast, prostate, lung, head and neck, and renal cancer, as well as neuroendocrine tumours, fibrosarcoma, glioma, glioblastoma, and neuroblastoma. The size of these tumours at start of treatment varied from being palpable to 200 mm^3^, but was not reported in a quarter of the studies.

Naturally following the variety of cancer types, a wide range of targets were used for TRT. These included PD-L1, integrin αvß3 and α4ß1, mesothelin, granzyme B, melanin, melanocortin 1 receptor (MC1R), huCD20, FAP, lipid rafts, macrophage mannose receptor (MMR), folate receptor, PSMA, carbonic anhydrase IX (CAIX), somatostatin receptor (SSTR), Cd11b, and poly ADP ribose polymerase (PARP). These antigens were targeted using mostly antibody- or peptide-based agents, but small molecules, nanobodies, and a tumour-selective alkylphosphocholine analog were also used. The majority of the targeting agents were labelled with beta-emitting radionuclides (Lu-177, Y-90, I-131) or alpha-emitting radionuclides (Ac-225, Bi-213, Th-227, At-211, Pb-212). Notably, one study used the combined beta- and Auger electron-emitter Cu-64, and one study aimed to investigate the biological effects of pure gamma-radiation using the diagnostic radionuclide Tc-99 m, although this study has been excluded from the meta-analyses due to various reasons. Tracers were mostly administered intravenously (23 studies) using different activities. Seven studies performed dosimetry and reported a tumour-absorbed dose varying from 1 to 29 Gy, 24 studies did not report a tumour-absorbed dose.

Various studies included multiple experiments comparing different ICI types. In total, 14 studies included aPD-L1, 11 aPD-1, five aCTLA-4, seven aPD-1 + aCTLA-4, and four aPD-L1 + aCTLA-4. Administration of ICIs included one to six treatments of 100–200 µg often given intraperitoneally (25 studies). TRT and ICI were mostly administered simultaneously, defined as treatment days overlapping or being maximally three days apart.

### Risk of bias assessment

**Fig. 3:**
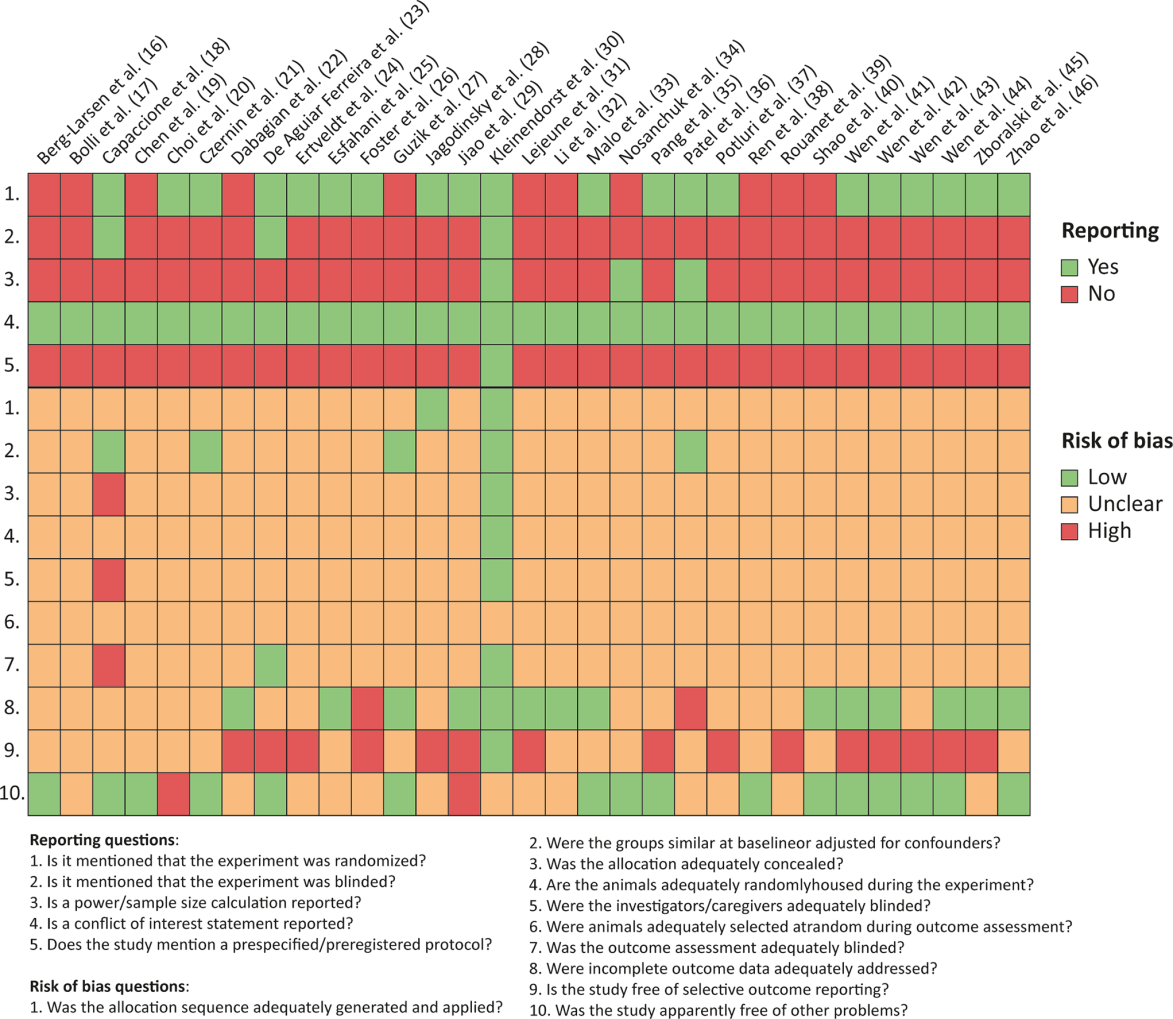
Risk of bias analysis

All included articles were assessed for presence of risks of bias (Fig. 3, Supplementary material 13). Five questions on reporting demonstrated that 20 studies mentioned randomization of the experiment, but only three mentioned blinding or sample size calculation. Conflict of interest statements were included in all studies, while only one study mentioned a preregistered protocol. These deficiencies in reporting important methodological details resulted in an unclear risk of bias for many domains of the risk of bias tool. Selective outcome reporting was scored as high risk for 15 studies, mainly because the follow-up time for which tumour size data were reported was often not consistent with the reported survival time, while these were the same animals. For two studies, high risk of bias was scored for other reasons, namely because the results of multiple independent experiments were combined. The comparisons of groups that clearly resulted from multiple experiments were excluded from the data analysis.

### Data analysis

**Fig. 4:**
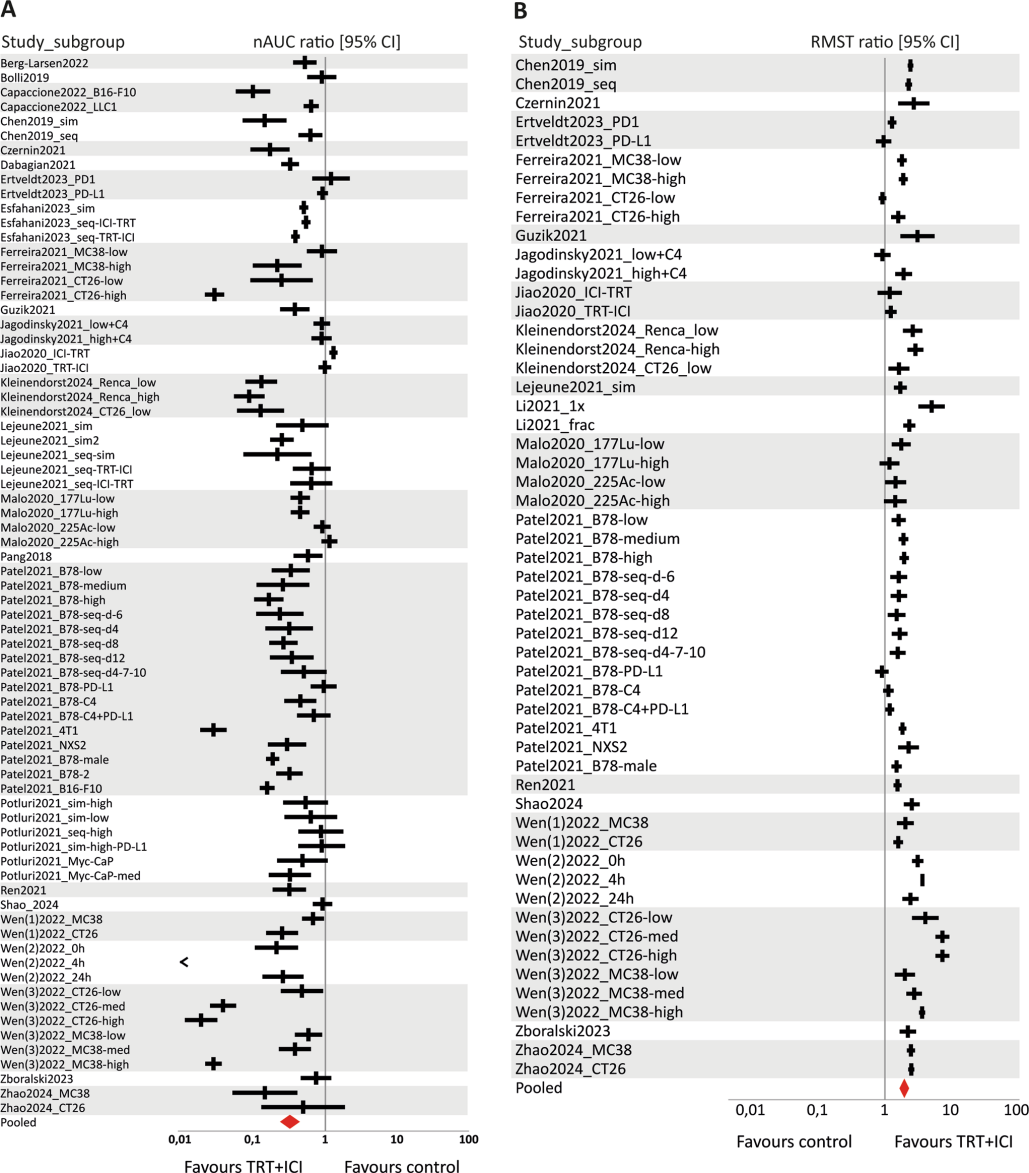
Forest plots of (**A**) nAUC ratios (tumour growth) and (**B**) RMST ratios (survival) with their 95% confidence interval (CI) of combined TRT/ICI treatment compared to untreated control. Favourable outcomes are indicated by a nAUC ratio below 1 and an RSMT ratio above 1. Statistical significance was reached when ratios with their 95% CI did not include the value of 1

**Fig. 5:**
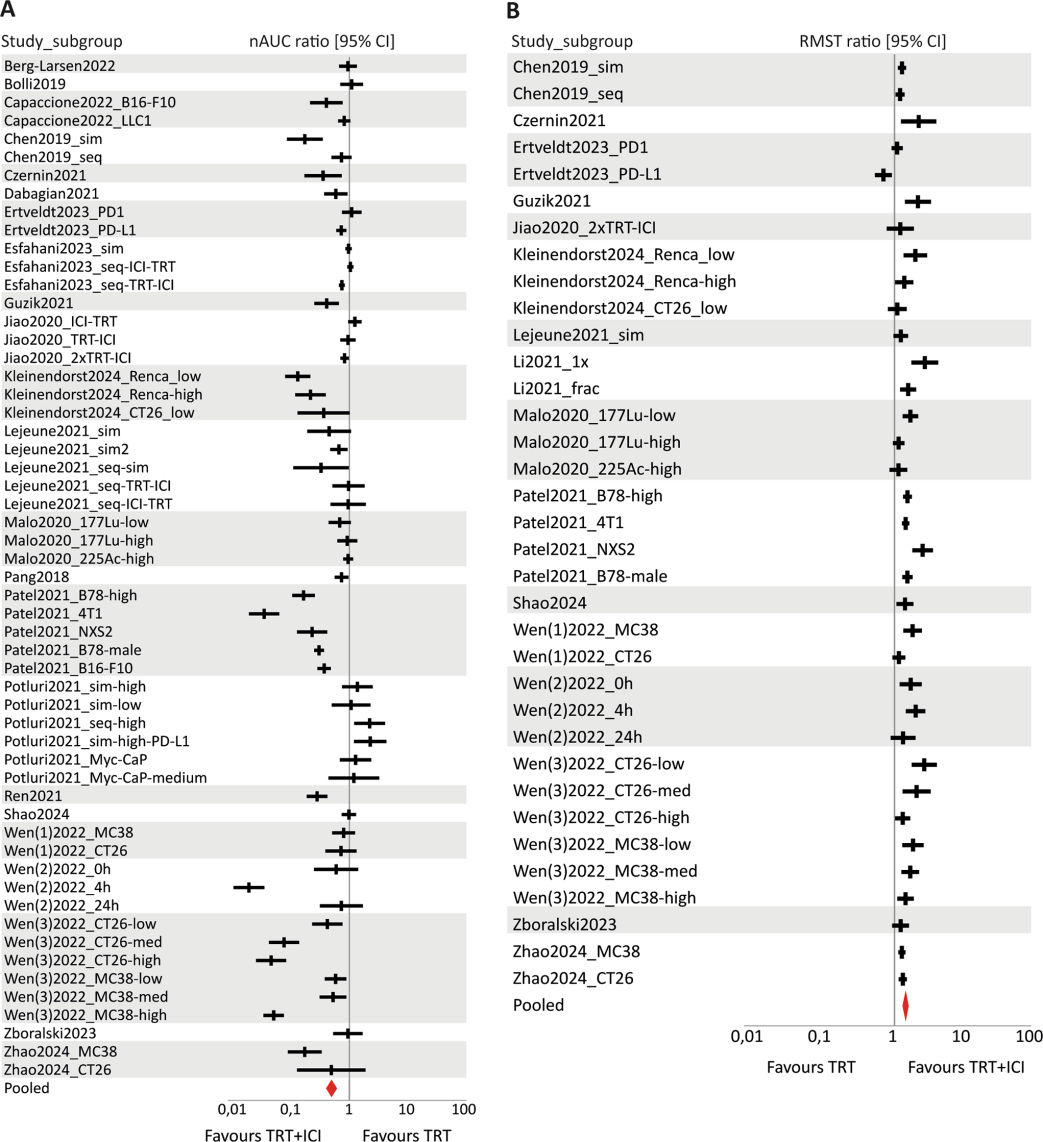
Forest plots of (**A**) nAUC ratios (tumour growth) and (**B**) RMST ratios (survival) with their 95% confidence interval (CI) of combined TRT/ICI treatment compared to TRT monotherapy. Favourable outcomes are indicated by a nAUC ratio below 1 and an RSMT ratio above 1. Statistical significance was reached when ratios with their 95% CI did not include the value of 1

**Fig. 6:**
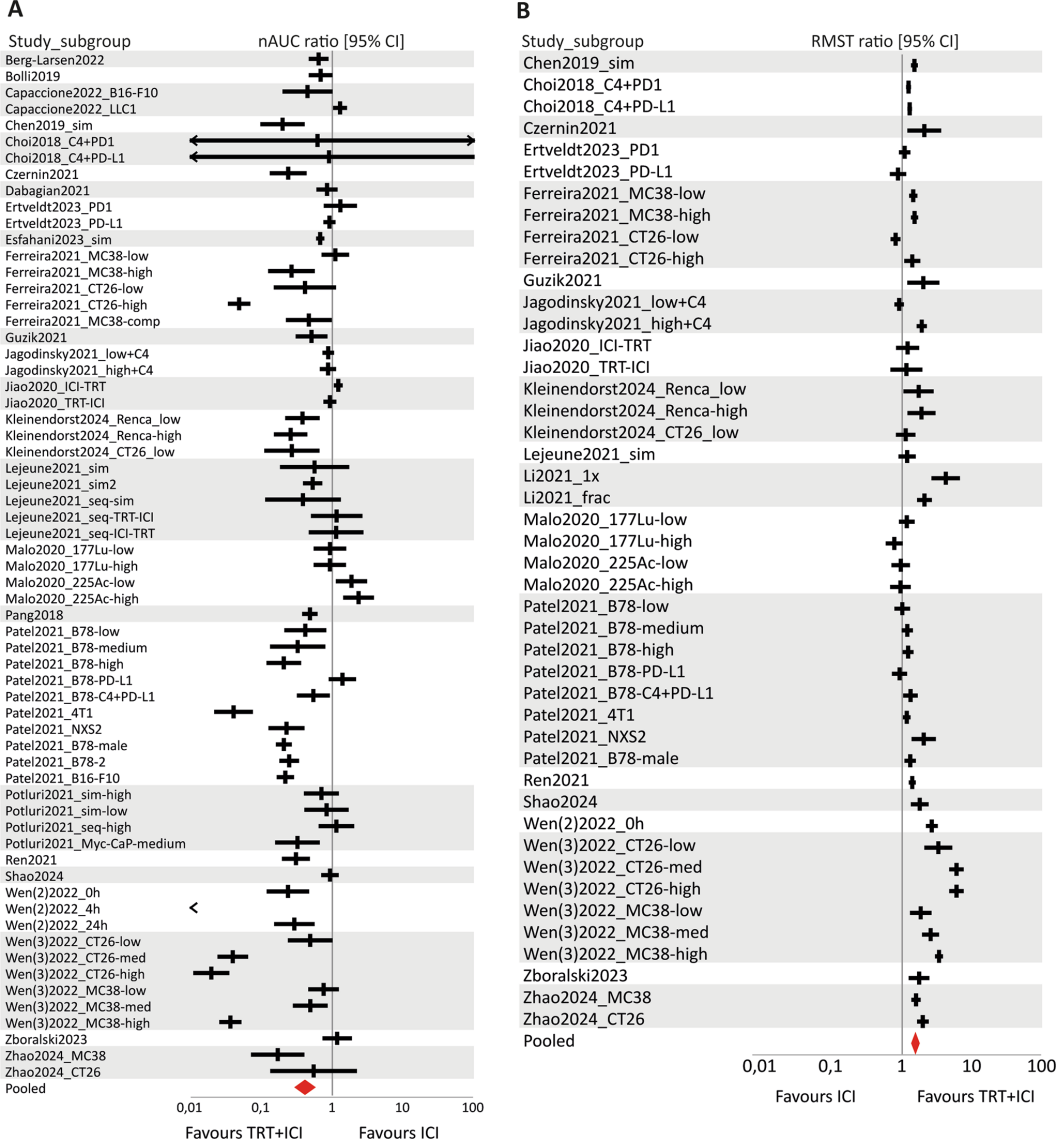
Forest plots of (**A**) nAUC ratios (tumour growth) and (**B**) RMST ratios (survival) with their 95% confidence interval (CI) of combined TRT/ICI treatment compared to ICI monotherapy. Favourable outcomes are indicated by a nAUC ratio below 1 and an RSMT ratio above 1. Statistical significance was reached when ratios with their 95% CI did not include the value of 1

The tumour growth (nAUC ratio) and survival (RMST ratio) data were combined in six separate forest plots, describing the effect of combined TRT/ICI on these two outcome measures compared to untreated control, TRT monotherapy, or ICI monotherapy (Figs. 4, 5 and 6).

#### Efficacy of combined TRT/ICI compared to untreated control

The data analysis included tumour growth data from 26 studies, comprising 73 comparisons and a total of 541 mice treated with combined TRT/ICI versus 327 untreated mice. For survival data, 19 studies were analysed, providing 54 comparisons between 465 treated mice and 267 untreated mice. The summary effect sizes of the combination treatment compared to untreated control were significantly in favour of the combination treatment for both tumour growth and survival, with a very high in-between study heterogeneity (nAUC ratio = 0.32 [0.25–0.42], I^2^ = 98.2% and RMST ratio = 1.96 [1.72–2.23], I^2^ = 98.1%) (Fig. 4).

#### Efficacy of combined TRT/ICI compared to TRT monotherapy

In evaluating combined TRT/ICI against TRT alone, 23 studies with tumour growth data were included, leading to 56 comparisons involving 445 TRT/ICI-treated mice and 341 TRT-treated mice. Survival outcomes from 16 studies covered 35 comparisons of 292 TRT/ICI-treated mice and 255 TRT-treated mice. The efficacy of combined TRT/ICI was also significantly in favour when compared to TRT monotherapy, and showed very high in-between study heterogeneity (nAUC ratio = 0.49 [0.41–0.59], I^2^ = 95.3% and RMST ratio = 1.44 [ 1.34–1.55], I^2^ = 76.7%) (Fig. 5).

#### Efficacy of combined TRT/ICI compared to ICI monotherapy

For comparisons between combined TRT/ICI and ICI alone, 25 studies provided tumour growth data. This comprised 63 comparisons involving 469 TRT/ICI-treated mice and 324 ICI-treated mice. Survival data from 19 studies were included, covering 45 comparisons of 368 TRT/ICI-treated mice and 266 ICI-treated mice. For both tumour growth and survival outcome data, the summary effect size was significantly in favour of the combination treatment compared to ICI monotherapy, again with very high in-between study heterogeneity (nAUC ratio = 0.41 [0.31–0.55], I^2^ = 97.9% and RMST ratio = 1.54 [1.38–1.72] I^2^ = 96.2%) (Fig. 6).

#### Summary of the effects including subgroup analyses

Heterogeneity was investigated by conducting subgroup analyses, of which results are summarized in Table 1. Predefined expected effect modulators included species, strain, housing type, age, sex, cancer type, tumour start size, radiation type, radionuclide, targeting molecule, type of ICI, and treatment schedule. Only subgroups with minimally ten independent studies within these categories were included in the analysis (Supplementary material 2). No differences in the overall outcomes were observed when analysing comparisons only including C57BL/6 mice, mice with an age of 6–8 weeks, female mice, a colon adenocarcinoma model, a tumour start size of 50–150 mm^3^, beta radiation, PD-L1 inhibitors, or a simultaneous treatment schedule. Regarding type of ICI treatment; subgroup analyses revealed that combined TRT/ICI therapy with aPD-L1 treatment reduced tumour growth significantly more compared to combined TRT/ICI therapy with aPD-1. This was the case for the comparisons of combined therapy versus TRT alone (*p* = 0.00009) and ICI alone (*p* = 0.048). Furthermore, the effect of combined TRT/ICI therapy with aPD-1 compared to TRT and ICI monotherapy appeared no longer to be effective (0.97 [0.75–1.25], 0.87 [0.52–1.44]).

### Sensitivity- and publication bias analyses

Sensitivity analysis for the post-hoc decision to estimate the standard error of nAUC ratio with two different methods was performed (Supplementary material 14). This revealed comparable results for both methods, indicating that our results for tumour growth are robust. To assess potential publication bias, funnel plots were examined for asymmetry, and Duval and Tweedie’s trim-and-fill method was applied to estimate the number of potentially missing studies. For tumour growth, the trim-and-fill analysis suggested missing studies on the left side of the funnel plot for all comparisons: 18 studies for combination versus control, 11 for combination versus TRT, and 17 for combination versus ICI. For survival, potential missing studies were identified on the right side of the plot for combination versus control (11 studies) and versus ICI (8 studies), while missing studies were found on the left side for combination versus TRT (7 studies). These findings suggest the presence of publication bias. However, adjustment of the overall effect size for the potential missing studies showed no changes in the direction of effects.

**Table 1:** Subgroup analysis for categories where data was available of ten or more independent studies

	Tumour growth								
Combination treatment versus	Control			TRT			ICI		
	nAUC ratio [95% CI]	*N*	I^2^	nAUC ratio [95% CI]	*N*	I^2^	nAUC ratio [95% CI]	*N*	I^2^
**Overall effect**	0.32 [0.25–0.42]	73	98.2	0.49 [0.41–0.59]	56	95.3	0.41 [0.31–0.55]	63	97.9
**Strain**: C57BL/6	0.35 [0.26–0.47]	47	98.0	0.49 [0.39–0.62]	32	94.5	0.44 [0.30–0.65]	41	98.1
**Age**: 6–8 weeks	0.32 [0.20–0.49]	28	99.1	N/A			0.37 [0.22–0.60]	25	99.0
**Sex**: female	0.29 [0.21–0.40]	44	97.5	0.35 [0.27–0.45]	27	95.5	0.36 [0.24–0.53]	38	96.4
**Cancer type**: colon adenocarcinoma	0.20 [0.13–0.30]	27	98.3	N/A			N/A		
**Tumour start size**: 50–150 mm^3^	0.29 [0.20–0.43]	36	98.9	0.40 [0.32–0.51]	34	95.1	0.38 [0.25–0.59]	33	98.6
**Radiation type**: beta	0.30 [0.24–0.38]	56	97.0	0.46 [0.37–0.57]	39	96.0	0.37 [0.28–0.48]	46	96.3
**Type of ICI**: aPD-1	0.61 [0.39–0.95]	21	95.6	0.97 [0.75–1.25] *	20	70.9	0.87 [0.52–1.44] *	18	86.5
**Type of ICI**: aPD-L1	0.26 [0.17–0.39]	25	98.9	0.37 [0.29–0.47] †	24	95.5	0.27 [0.17–0.44] †	21	98.8
**Treatment schedule**: simultaneous	0.32 [0.22–0.45]	45	98.6	0.52 [0.42–0.65]	45	94.5	0.43 [0.30–0.62]	43	98.3
	**Survival**								
**Combination treatment versus**	**Control**			**TRT**			**ICI**		
	**RMST ratio [95% CI]**	**N**	**I** ^**2**^	**RMST ratio [95% CI]**	**N**	**I** ^**2**^	**RMST ratio [95% CI]**	**N**	**I** ^**2**^
**Overall effect**	1.96 [1.72–2.23]	54	98.1	1.44 [ 1.34–1.55]	35	76.7	1.54 [1.38–1.72]	45	96.2
**Strain**: C57BL/6	1.90 [1.62–2.23]	35	98.2	1.42 [1.30–1.55]	20	78.2	1.51 [1.32–1.74]	27	95.7
**Sex**: female	1.93 [1.65–2.25]	35	97.2	N/A			1.59 [1.38–1.84]	28	97.1
**Tumour start size**: 50–150 mm^3^	2.32 [2.04–2.64]	34	97.2	1.45 [ 1.35–1.56]	29	67.1	1.80 [1.57–2.06]	29	96.4
**Radiation type**: beta	1.96 [1.73–2.23]	41	96.8	1.47 [ 1.36–1.59]	24	71.9	1.57 [1.39–1.78]	34	96.8
**Type of ICI**: aPD-L1	2.58 [2.21–3.01]	21	98.1	N/A			N/A		
**Treatment schedule**: simultaneous	2.39 [2.08–2.74]	31	97.5	1.42 [1.32–1.54]	30	75.0	1.76 [1.53–2.02]	29	97.0

RMST ratio with 95% confidence interval, N represents the number of comparisons in the data analysis, I^2^ was used as a measure of heterogeneity, N/A = not applicable because subgroup analysis could not be performed due to insufficient number of studies. * indicates non-significant effect size. † indicates significant difference with aPD-1 subgroup

## Discussion

In this systematic review we present an overview of the existing literature on the therapeutic efficacy of combined TRT/ICI treatment in animal tumour models. Our analysis of 31 studies revealed considerable variability in study design, including differences in animal model (e.g. sex, age, cancer type), TRT treatment parameters (e.g. target, radionuclide), and ICI type. Despite this heterogeneity, the overall meta-analysis indicates that combined TRT/ICI treatment is more effective in reducing tumour growth and prolonging survival than either TRT or ICI monotherapies. Subgroup analysis further suggests that the type of ICI influences treatment outcomes, with combination therapy using aPD-1 being less effective than that using aPD-L1. However, the current literature was insufficient to perform subgroup analyses for most parameters of interest.

This review represents the first comprehensive overview of the available preclinical literature on combined TRT/ICI combination therapies. It provides important evidence supporting the efficacy of this combination treatment across various oncological indications, although its generalizability to clinical settings is limited by inherent model constraints. All included studies were conducted in mice, likely due to their genetic similarity to humans and practical benefits such as availability and low cost [47]. Notably, in this review it became transparent that 20 (64.5%) of the included studies involved mice younger than eight weeks old, despite the fact that mature T cell responses require animals of at least eight weeks of age [48]. Moreover, most studies used syngeneic mouse models with subcutaneously injected tumours. While these models are practical and reproducible, they often lack the complexity of the TME seen in human cancers. The rapid tumour growth in these models results in a small therapeutic window, limiting the ability to evaluate optimal treatment schedules, with the majority of studies investigating simultaneous rather than sequential approaches. More clinically relevant models – such as metastatic or orthotopic tumours, genetically engineered mouse models (GEMMs) with prolonged tumour development, and humanized patient-derived xenografts – could better capture this complexity. These models are particularly well suited for evaluating systemic treatments like combined TRT/ICI, especially when targeting TME-associated factors like FAP [49], optimizing treatment schedules, and assessing clinical radiopharmaceuticals directed at human targets [49]. To ensure robust clinical translatability of effective TRT/ICI combination therapy, future preclinical research should be broadened to include diverse models, using appropriately aged animals and well-characterized tumours that more accurately reflect the human TME. To date, animal studies remain essential due to the complexity of the immune system which cannot be fully captured in in vitro models. However, the integration of sophisticated in vitro models, such as 3D and co-culture systems, could complement preclinical research (e.g. exploring specific components of the immune-related dose-response relationships) and help reduce reliance on animal studies.

With this review and analysis, we further aimed to identify treatment parameters influencing outcomes, to guide future clinical trial design. Our subgroup analysis revealed that some of the heterogeneity in the tumour growth outcomes can be attributed to ICI type, with TRT combined with aPD-1 showing lower efficacy than when combined with aPD-L1. However, our analysis was limited to these two agents, as the available data did not allow for subgroup evaluations of aCTLA-4 or their dual-combinations. While current preclinical research has focused primarily on these clinically established ICIs, which have broad clinical relevance across many cancer types, the emergence of novel targets such as lymphocyte activation gene-3, T cell immunoglobulin and ITIM domain (TIGIT), T cell immunoglobulin and mucin-domain containing-3 (TIM-3), and indoleamine 2,3-dioxygenase (IDO) calls for further investigation to evaluate their potential synergy with TRT [5]. Regarding TRT treatment parameters, radiation type and tumour-absorbed dose are key factors likely to influence therapeutic outcomes. However, due to the limited number of studies investigating alpha-emitters, we could not perform a subgroup analysis comparing the outcomes for alpha and beta radiation. Notably, only one study directly compared these two types of radiation [33]. Additionally, to facilitate better comparisons across radiation types and studies, incorporating preclinical dosimetry is essential [50]. Here, only 7 (22.6%) of the studies reported dosimetry data, highlighting a significant gap in our understanding of dose-response relationships in combined TRT/ICI treatment and the need for dosimetry to derive clinically relevant dosing estimates [51]. Although the optimal TRT tumour-absorbed dose for effective immune priming remains to be determined, our findings suggest that reducing TRT activity may be feasible in combination with ICI, as combination treatment demonstrated more therapeutic benefit compared to TRT monotherapy at the same activity.

Current trials largely adhere to standard treatment regiments that are based on the maximum-tolerated dose. However, given the frequent concern of TRT-induced haematological toxicity, the evaluation of low subtherapeutic radiation activities in combination with ICI is recommended to investigate in clinical trials. Furthermore, it will be essential to study treatment sequencing more systematically. For example, a recent phase 1 clinical trial investigating a single dose of [^177^Lu]Lu-PSMA-617 followed by maintenance pembrolizumab in metastatic castration-resistant prostate cancer demonstrated that this sequence was safe and feasible, with preliminary signs of anti-tumour efficacy [8]. Additionally, this study incorporated blood collections and metastatic tumour biopsies to assess immune cell dynamics following treatment, revealing differences in certain circulating immune cell subsets (e.g. CD8^+^ effector cells and natural killer T cells) between responding and non-responding patients. Such immune profiling is crucial to better understand the immunological effects of TRT and combination treatment, and will help inform preclinical studies to further optimize treatment strategies.

Despite these valuable insights, this study has some limitations that should be considered when interpreting the findings. First, between-study heterogeneity was high, which is expected in animal research due to its often exploratory nature. To account for this, we applied a random-effects model, performed sensitivity analyses, and investigated potential sources of heterogeneity through subgroup analyses. Second, the reliability of our results depends on the quality of the included studies, and poor reporting in animal studies remains a significant limitation. As highlighted by our risk of bias analysis, the quality of reporting is still insufficient, despite the availability of the ARRIVE guidelines since 2010 [52]. We strongly encourage adherence to these guidelines and the recently published recommendation for reporting preclinical radiobiological studies in TRT [53], along with preregistration of protocols in databases like animalstudyregistry.org [54] or preclinicaltrials.eu [55] to improve transparency and reproducibility. Additionally, including an animal characteristics table for each experiment, similar to clinical studies, would improve reporting. While certain standards, such as blinding, are well established in clinical research, they are not yet universally applied in preclinical research due to practicalities, but this should be addressed to enhance study quality and translational value. Third, in this review we showed the possible presence of publication bias, which may point to inflated effect sizes, because studies showing neutral or negative results may be missing. Fourth, while conducting this review we experienced that that extracting outcome data was very challenging. The lack of standardized reporting of tumour growth contributed to variability in nAUC ratios. Inconsistent tumour measurement methods, reporting of individual data versus group means, and poor reporting on how those means are calculated added to this variability. In addition, outcome data were frequently not made available following the FAIR principles (findable, accessible, interoperable and reusable [56]),. As a result, data was frequently extracted manually from graphical representation, increasing the risk of inaccuracies, or was not available at all, even after direct requests to the authors. Fifth, it should be emphasized that although nAUC was considered the most suitable outcome parameter, it has its limitations, including loss of information and reduced detection power (see also Supplementary material 1.4). Similarly, while RMST ratio was preferred for survival analysis, it also comes with challenges. Low event rates, unclear reasons for animal sacrifice, and unsubstantiated follow-up times complicate the interpretation of survival outcomes.

## Conclusion

This is the first systematic review providing a comprehensive meta-analysis of preclinical studies investigating the therapeutic efficacy of combined TRT/ICI treatment in tumour models. The findings strongly support the therapeutic potential of combined TRT/ICI, which should be verified in well-designed clinical trials. To refine optimal treatment strategies, preclinical studies remain essential to systematically evaluate the impact of treatment parameters (e.g. ICI type, radionuclide, tumour-absorbed radiation dose, treatment sequencing). Future research should prioritize the use of clinically relevant preclinical models, integrate dosimetry to facilitate cross-study comparisons and clinical translation of effective and safe absorbed doses, and adhere to robust reporting guidelines. A summary of the key findings from this systematic review based on the included studies and recommendations for future research is provided in Table 2.

**Table 2:** Summary of key findings from systematic review and recommendations for future research on combined TRT/ICI

Aspect	Key findings from systematic review based on included studies	Recommendations for future research
Therapeutic efficacy	Combined TRT/ICI is more effective in reducing tumour growth and prolonging survival than no treatment or monotherapy.	Confirm safety and efficacy in prospective (randomized) clinical trials.
Study characteristics	Efficacy is being investigated across a variety of mouse models (sex, age, cancer type) and TRTs (target, radionuclides).	Use of appropriate and clinically relevant preclinical models (e.g. age ≥ 8 weeks, metastatic/orthotopic tumours, clinically relevant targets) to further evaluate key treatment parameters.
Reporting quality	Most studies lack detailed methodological reporting, preventing a thorough assessment of risk of bias and study quality.	Improve transparency through protocol preregistration and standardized reporting (e.g. ARRIVE guidelines and the recommendations for reporting preclinical radiobiological studies in TRT [53]).
TRT activity and tumour-absorbed dose	Reducing TRT activity may be feasible in combination with ICI, given its benefit over TRT monotherapy. No tumour-absorbed dose is reported in the majority of studies.	Evaluate subtherapeutic radiation activities in combination with ICI in clinical trials. Incorporate dosimetry in preclinical and clinical studies.
Radionuclide selection	Direct comparisons between radiation types or radionuclides in sub-analyses could not be performed due to an insufficient number of independent studies, while there is one individual study making this comparison [33].	Systematic evaluation of radiobiological effects of different radiation types and radionuclides in preclinical models.
ICI type	Sub-analysis on studies combining TRT with aPD-1 did not demonstrate a significant effect on tumour growth, while individual studies do [16, 21, 22, 24, 33, 37]. Direct comparisons between ICI types in a sub-analysis could not be performed due to an insufficient number of independent studies, while there are individual studies making this comparison [20, 23, 28, 36, 37, 39].	Expand the evidence base with well-controlled studies comparing different ICI types.
Treatment sequencing	Direct comparisons between different timings of TRT and ICI administration in a sub-analysis could not be performed due to an insufficient number of independent studies, while there are individual studies making this comparison [19, 24, 31, 32, 36, 37, 42, 44].	Investigate treatment timing in suitable preclinical models (e.g. slow tumour growth dynamics) and clinical studies.
Mechanistic insights	Not included in this systematic review.	Include immune cell profiling of blood and tumour tissues in (pre)clinical studies (like [8]) to 1) identify key mechanisms, immune cell subsets driving treatment response, and their dynamics; 2) guide (pre)clinical research toward more targeted and effective strategies.

## Electronic supplementary material

Below is the link to the electronic supplementary material.

Supplementary Material 1

Supplementary Material 2

Supplementary Material 3

Supplementary Material 4

Supplementary Material 5

Supplementary Material 6

Supplementary Material 7

Supplementary Material 8

Supplementary Material 9

Supplementary Material 10

Supplementary Material 11

Supplementary Material 12

Supplementary Material 13

Supplementary Material 14

Supplementary Material 15

## Data Availability

All data generated or analysed during this study are included in this published article and its supplementary files.

## Abbreviations

AUC: Area under the curve
CAF: Cancer-associated fibroblast
CAIX: Carbonic anhydrase IX
CTLA-4: Cytotoxic T-lymphocyte associated protein-4
EBRT: External beam radiation therapy
FAP: Fibroblast activation protein
GEMM: Genetically engineered mouse model
ICI: Immune checkpoint inhibitor
IDO: Indoleamine 2,3-dioxygenase
LAG-3: Lymphocyte activation gene-3
MC1R: Melanocortin 1 receptor
MMR: Macrophage mannose receptor
nAUC: Normalized area under the curve
PARP: Poly ADP ribose polymerase
PD-1: Programmed cell death-1
PD-L1: Programmed death ligand-1
PRISMA: Preferred reporting items for systematic reviews and meta-analyses
PSMA: Prostate-specific membrane antigen
RMST: Restricted mean survival time
SSTR: Somatostatin receptor
TIGIT: T cell immunoglobulin and ITIM domain
TIM-3: T cell immunoglobulin and mucin-domain containing-3
TME: Tumour microenvironment
TRT: Targeted radionuclide therapy
