# Supplementary material for: Efficacy of combined targeted radionuclide therapy and immune checkpoint Inhibition in animal tumour models: a systematic review and meta-analysis of the literature
Source: Eur J Nucl Med Mol Imaging. 2025 Apr 26;52(12):4735–51. doi: 10.1007/s00259-025-07293-0 (PMC12491095; doi:10.1007/s00259-025-07293-0)
Supplement: Supplementary file 1 — Supplementary Material 1 [file 259_2025_7293_MOESM1_ESM.docx]

**SUPPLEMENTAL MATERIAL**

**1 Search strategy**

**1.1 MEDLINE (via PubMed)**

**Search component 1: non-human animal species**
Animal search filter described by Mierden et al. [1]

AND

**Search component 2: cancer**
"Neoplasms"[MeSH Terms] OR "Neoplasm"[tiab] OR "Neoplasms"[tiab] OR "Neoplasia"[tiab] OR "Neoplasias"[tiab] OR "Tumor"[tiab] OR "Tumour"[tiab] OR "Tumors"[tiab] OR "Tumours"[tiab] OR "Antitumor"[tiab] OR "Antitumour"[tiab] OR "Anticancer"[tiab] OR "Cancer"[tiab] OR "Cancers"[tiab] OR "Cancerous"[tiab] OR "Malignant"[tiab] OR "Malignancy"[tiab] OR "Malignancies"[tiab] OR "Carcinoma"[tiab] OR "Carcinomas"[tiab] OR "Adenocarcinoma"[tiab] OR "Leukemia"[tiab] OR "Leukemias"[tiab] OR "Lymphoma"[tiab] OR "Lymphomas"[tiab] OR "Melanoma"[tiab] OR "Melanomas"[tiab] OR "Myeloma"[tiab] OR "Myelomas"[tiab] OR "Sarcoma"[tiab] OR "Sarcomas"[tiab] OR "Leiomyosarcoma"[tiab] OR "Chondrosarcoma"[tiab] OR "blastoma"[tiab] OR "blastomas"[tiab] OR "Retinoblastoma"[tiab] OR "Neuroblastoma"[tiab] OR "Medulloblastoma"[tiab] OR "Nephroblastoma"[tiab] OR "Hepatoblastoma"[tiab] OR "Glioblastoma"[tiab] OR "Glioma"[tiab] OR "Meningioma"[tiab] OR "Mesothelioma"[tiab]

AND

**Search component 3: targeted radionuclide therapy**
"Radioisotopes"[MeSH Terms] OR "Radiopharmaceuticals"[MeSH Terms] OR "Radiopharmaceuticals"[pharmacological action] OR "Radionuclide*"[tiab] OR "Radio-nuclide*"[tiab] OR "Radioligand*"[tiab] OR "Radio-ligand*"[tiab] OR "Radioisotope*"[tiab] OR "Radio-isotope*"[tiab] OR "Radioactive isotope*"[tiab] OR "Radiopeptide*"[tiab] OR "Radio-peptide" OR "Radiopharmaceutical*"[tiab] OR "Targeted radiotherap*"[tiab] OR "Molecular radiotherap*"[tiab] OR "Alpha-emitt*"[tiab] OR "Alpha particle emit*" OR "Targeted alpha therap*"[tiab] OR "Targeted alpha particle therap*"[tiab] OR "Targeted alpha-emitter therap*"[tiab] OR "Targeted alpha-radiation therap*"[tiab] OR "Beta-emitt*"[tiab] OR "Beta particle emit*"[tiab] OR "Auger-emitt*"[tiab] OR "iodine 131"[Supplementary Concept] OR "Iodine-131"[tiab] OR "131I"[tiab] OR "I-131"[tiab] OR "yttrium 90"[Supplementary Concept] OR "Yttrium-90"[tiab] OR "90Y"[tiab] OR "Y-90"[tiab] OR "lutetium 177"[Supplementary concept] OR "Lutetium-177"[tiab] OR "177Lu"[tiab] OR "Lu-177"[tiab] OR "radium 223"[Supplementary concept] OR "Radium-223"[tiab] OR "223Ra"[tiab] OR "Ra-223"[tiab] OR "Radium-224"[tiab] OR "224Ra"[tiab] OR "Ra-224"[tiab] OR "actinium 225"[Supplementary concept] OR "Actinium-225"[tiab] OR "225Ac"[tiab] OR "Ac-225"[tiab] OR "bismuth 213"[Supplementary concept] OR "Bismuth-213"[tiab] OR "213Bi"[tiab] OR "Bi-213"[tiab] OR "astatine 211"[Supplementary concept] OR "Astatine-211"[tiab] OR "211At"[tiab] OR "At-211"[tiab] OR "lead 212"[Supplementary concept] OR "Lead-212"[tiab] OR "212Pb"[tiab] OR "Pb-212"[tiab] OR "terbium 161"[Supplementary concept] OR "Terbium-161"[tiab] OR "161Tb"[tiab] OR "Tb-161"[tiab] OR "thorium 227"[Supplementary concept] OR "Thorium-227"[tiab] OR "227Th"[tiab] OR "Th-227"[tiab] OR "samarium 153"[Supplementary concept] OR "Samarium-153"[tiab] OR "153Sm"[tiab] OR "Sm-153"[tiab] OR "phosphorus 32"[Supplementary concept] OR "Phosphorus-32"[tiab] OR "32P"[tiab] OR "P-32"[tiab] OR "rhenium 186"[Supplementary concept] OR "Rhenium-186"[tiab] OR "186Re"[tiab] OR "Re-186"[tiab] OR "rhenium 188"[Supplementary concept] OR "Rhenium-188"[tiab] OR "188Re"[tiab] OR "Re-188"[tiab] OR "scandium 47"[Supplementary concept] OR "Scandium-47"[tiab] OR "47Sc"[tiab] OR "Sc-47"[tiab] OR "copper 67"[Supplementary concept] OR "Copper-67"[tiab] OR "67Cu"[tiab] OR "Cu-67"[tiab]

AND

**Search component 4: immune checkpoint inhibition**
"Immune Checkpoint Inhibitors"[MeSH Terms] OR "Immune Checkpoint Inhibitors" [Pharmacological Action] OR "Immune Checkpoint*"[tiab] OR "Checkpoint inhibitor*"[tiab] OR "Anti-PD-L1"[tiab] OR "Anti-PDL1"[tiab] OR "PD-L1"[tiab] OR "PDL1"[tiab] OR "CD274"[tiab] OR "B7-H1"[tiab] OR "B7H1"[tiab] OR "Programmed Death-Ligand 1"[tiab] OR "Anti-PD-1"[tiab] OR "Anti-PD1"[tiab] OR "PD-1"[tiab] OR "PD1"[tiab] OR "PDCD1"[tiab] OR "CD279"[tiab] OR "Programmed Cell Death Protein 1"[tiab] OR "Anti-CTLA-4"[tiab] OR "Anti-CTLA4"[tiab] OR "CTLA-4"[tiab] OR "CTLA4"[tiab] OR "CD152"[tiab] OR "Cytotoxic T-Lymphocyte-Associated Protein 4"[tiab] OR "Cytotoxic T-Lymphocyte Protein 4"[tiab] OR "Atezolizumab"[tiab] OR "Avelumab"[tiab] OR "Cemiplimab"[tiab] OR "Durvalumab"[tiab] OR "Ipilimumab"[tiab] OR "Nivolumab"[tiab] OR "Pembrolizumab"[tiab] OR "Tremelimumab"[tiab] OR "Spartalizumab"[tiab] OR "Tecentriq"[tiab] OR "Bavencio"[tiab] OR "Libtayo"[tiab] OR "Imfinzi"[tiab] OR "Yervoy"[tiab] OR "Opdivo"[tiab] OR "Keytruda"[tiab] OR "atezolizumab"[Supplementary Concept] OR "avelumab"[Supplementary Concept] OR "cemiplimab"[Supplementary Concept] OR "durvalumab"[Supplementary Concept] OR "ipilimumab"[MeSH Terms] OR "nivolumab"[MeSH Terms] OR "pembrolizumab"[Supplementary Concept] OR "tremelimumab"[Supplementary Concept] OR "spartalizumab"[Supplementary Concept] OR "Immune Checkpoint Proteins"[MeSH Terms] OR "B7-2"[tiab] OR "B70"[tiab] OR "CD86"[tiab] OR "CD40"[tiab] OR "TNFRSF5"[tiab] OR "CDw40"[tiab] OR "Lymphocyte Activation Gene 3"[tiab] OR "CD223"[tiab] OR "LAG-3"[tiab] OR "OX40"[tiab] OR "CD134"[tiab] OR "TIGIT"[tiab] OR "ICOS"[tiab] OR "PD-L2"[tiab] OR "PDL2"[tiab] OR "CD273"[tiab] OR "B7-DC"[tiab] OR "B7DC"[tiab]

**1.2 EMBASE (via Ovid)**

**Search component 1: non-human animal species**
Animal search filter described by Mierden et al. [1]

AND

**Search component 2: cancer**
Exp Neoplasm/ OR ("Neoplasm" or "Neoplasms" or "Neoplasia" or "Neoplasias" or "Tumor" or "Tumour" or "Tumors" or "Tumours" or "Antitumor" or "Antitumour" or "Anticancer" or "Cancer" or "Cancers" or "Cancerous" or "Malignant" or "Malignancy" or "Malignancies" or "Carcinoma" or "Carcinomas" or "Adenocarcinoma" or "Leukemia" or "Leukemias" or "Lymphoma" or "Lymphomas" or "Melanoma" or "Melanomas" or "Myeloma" or "Myelomas" or "Sarcoma" or "Sarcomas" or "Leiomyosarcoma" or "Chondrosarcoma" or "blastoma" or "blastomas" or "Retinoblastoma" or "Neuroblastoma" or "Medulloblastoma" or "Nephroblastoma" or "Hepatoblastoma" or "Glioblastoma" or "Glioma" or "Meningioma" or "Mesothelioma").ti,ab,kf.

AND

**Search component 3: targeted radionuclide therapy**
exp radioisotope/ or exp Radiopharmaceutical agent/ or terbium/ or ("Radionuclide*" or "Radio-nuclide*" or "Radioligand*" or "Radio-ligand*" or "Radioisotope*" or "Radio-isotope*" or "Radioactive isotope*" or "Radiopeptide*" or "Radio-peptide" or "Radiopharmaceutical*" or "Targeted radiotherap*" or "Molecular radiotherap*" or "Alpha-emitt*" or "Alpha particle emit*" or "Targeted alpha therap*" or "Targeted alpha particle therap*" or "Targeted alpha-emitter therap*" or "Targeted alpha-radiation therap*" or "Beta-emitt*" or "Beta particle emit*" or "Auger-emitt*" or "Iodine-131" or "131I" or "I-131" or "Yttrium-90" or "90Y" or "Y-90" or "Lutetium-177" or "177Lu" or "Lu-177" or "Radium-223" or "223Ra" or "Ra-223" or "Radium-224" or "224Ra" or "Ra-224" or "Actinium-225" or "225Ac" or "Ac-225" or "Bismuth-213" or "213Bi" or "Bi-213" or "Astatine-211" or "211At" or "At-211" or "Lead-212" or "212Pb" or "Pb-212" or "Terbium-161" or "161Tb" or "Tb-161" or "Thorium-227" or "227Th" or "Th-227" or "Samarium-153" or "153Sm" or "Sm-153" or "Phosphorus-32" or "32P" or "P-32" or "Rhenium-186" or "186Re" or "Re-186" or "Rhenium-188" or "188Re" or "Re-188" or "Scandium-47" or "47Sc" or "Sc-47" or "Copper-67" or "67Cu" or "Cu-67").ti,ab,kf.

AND

**Search component 4: immune checkpoint inhibition**

Immune Checkpoint Inhibitor/ or atezolizumab/ or avelumab/ or cemiplimab/ or durvalumab/ or ipilimumab/ or nivolumab/ or pembrolizumab/ or ticilimumab/ or spartalizumab/ or Immune Checkpoint Protein/or ("Immune Checkpoint*" or "Checkpoint inhibitor*" or "Anti-PD-L1" or "Anti-PDL1" or "PD-L1" or "PDL1" or "CD274" or "B7-H1" or "B7H1" or "Programmed Death-Ligand 1" or "Anti-PD-1" or "Anti-PD1" or "PD-1" or "PD1" or "PDCD1" or "CD279" or "Programmed Cell Death Protein 1" or "Anti-CTLA-4" or "Anti-CTLA4" or "CTLA-4" or "CTLA4" or "CD152" or "Cytotoxic T-Lymphocyte-Associated Protein 4" or "Cytotoxic T-Lymphocyte Protein 4" or "Atezolizumab" or "Avelumab" or "Cemiplimab" or "Durvalumab" or "Ipilimumab" or "Nivolumab" or "Pembrolizumab" or "Tremelimumab" or "Spartalizumab" or "Tecentriq" or "Bavencio" or "Libtayo" or "Imfinzi" or "Yervoy" or "Opdivo" or "Keytruda" or "B7-2" or "B70" or "CD86" or "CD40" or "TNFRSF5" or "CDw40" or "Lymphocyte Activation Gene 3" or "CD223" or "LAG-3" or "OX40" or "CD134" or "TIGIT" or "ICOS" or "PD-L2" or "PDL2" or "CD273" or "B7-DC" or "B7DC").ti,ab,kf.

**2 Inclusion and exclusion criteria**

|  | Inclusion | Exclusion |
| --- | --- | --- |
| Type of study (design) | Original controlled experimental animal study | Non-original research (e.g. review, poster, conference report)  Study without control group |
| Type of animals/population | Experimental animals with tumours | No animal model  No tumour model |
| Type of intervention | Targeted radionuclide therapy + immune checkpoint inhibition | Other type of radiotherapy Other type of immunotherapy No combination therapy |
| Outcome measures | Tumour growth data Survival data | No tumour growth nor survival data |

Examples of exclusion are studies that included combination therapies of ICIs with free radionuclides, such as Ra-223 [2, 3] or with radiolabelled non-targeted molecules or nanoparticles [4-11], since we defined TRT as a systemic treatment which uses an antigen-targeting molecule labelled with a radionuclide [12]. Additionally, one study mentioned a combination therapy experiment, but data were not shown and could not be obtained by contacting the authors, and was therefore excluded [13].

**3 Data extraction assumptions**

For determination of the sample size (n) per timepoint for the tumour growth data, the following assumptions were made during data extraction. If survival data was not available, it was assumed that all animals survived until the end of follow-up. If the article stated a range for sample size (e.g. n=7-9), and the exact number could not be derived from the text or survival data, the largest sample size was assumed. Furthermore, it was assumed that tumour size measurements were done on the day an animal was sacrificed, and thus this animal was taken along in the sample size of the timepoint of sacrifice. Regarding the extraction of mean and variation in tumour size per timepoint, if it was not reported what variation was depicted in the graph, it was assumed that the extracted value was SEM.

**4 Data analysis**

For survival data, the RMST ratio was used in the current study, rather than the commonly used hazard ratio. RMST has been suggested as an alternative measure in survival analyses when the proportional hazards assumption is not met, or when the event rate is low [14]. In real-world data, both clinical and preclinical, the proportional hazard assumption is rarely achieved. Additionally, in preclinical studies the sample size is often small and treatment may be very effective, both resulting in a low event rate. This also makes median survival as outcome hardly calculable. Therefore, using Cox regression to obtain hazard ratios is not appropriate to assess survival data of preclinical studies.

For tumour growth data, different outcome measures are generally used for statistical analyses, e.g. tumour volume at a specific timepoint, tumour doubling time, or median time to reach a certain threshold [15]. This inconsistency makes it challenging to analyse tumour growth data in a meta-analysis, especially since the outcome of choice may depend on the tumour model. For example, tumour doubling time may not be appropriate for a fast-growing model that already reaches this time before a treatment becomes effective, and similarly median time to reach a threshold may not be reached at all in a model in which humane endpoints such as ulceration or illness are reached earlier. The outcome measure nAUC can be applied for all tumour growth curves independent of the tumour model, and includes data over the complete course of the study while accounting for the lifetime of animals in each group. However, it is important to note that this measure may not always demonstrate between-group differences, because the same nAUC may result from curves with different shapes that would be characterized as different according to a different measure. Furthermore, the power of nAUC as measure is generally limited in detecting small effect sizes. Still, for the data included in this meta-analysis originating from a variety of tumour models, nAUC ratio was considered the best outcome measure.

**REFERENCES**

1. van der Mierden, S., et al., *Laboratory animals search filter for different literature databases: PubMed, Embase, Web of Science and PsycINFO.* Laboratory Animals, 2022. **56**(3): p. 279-286.

2. Saylor, P.J., et al., *The radiopharmaceutical radium-223 has immunomodulatory effects in patients and facilitates anti-programmed death receptor-1 therapy in murine models of bone metastatic prostate cancer.* Radiotherapy and Oncology, 2024. **192**: p. 110091.

3. Vardaki, I., et al., *Radium-223 Treatment Increases Immune Checkpoint Expression in Extracellular Vesicles from the Metastatic Prostate Cancer Bone Microenvironment.* Clin Cancer Res, 2021. **27**(11): p. 3253-3264.

4. Vito, A., et al., *Combined radionuclide therapy and immunotherapy for treatment of triple negative breast cancer.* International Journal of Molecular Sciences, 2021. **22**.

5. Zhang, J., et al., *Alpha radionuclide-chelated radioimmunotherapy promoters enable local radiotherapy/chemodynamic therapy to discourage cancer progression.* Biomaterials Research, 2022. **26**.

6. Mare, S.D., et al., *Diffusing Alpha-Emitters Radiation Therapy Promotes a Proimmunogenic Tumour Microenvironment and Synergizes With Programmed Cell Death Protein 1 Blockade.* Int J Radiat Oncol Biol Phys, 2023. **115**(3): p. 707-718.

7. Zhang, J., et al., *Biomimetic radiosensitizers unlock radiogenetics for local interstitial radiotherapy to activate systematic immune responses and resist tumour metastasis.* Journal of Nanobiotechnology, 2022. **20**(1): p. 103.

8. Cai, Z., et al., *(90)Y-Labeled Gold Nanoparticle Depot (NPD) Combined with Anti-PD-L1 Antibodies Strongly Inhibits the Growth of 4T1 Tumours in Immunocompetent Mice and Induces an Abscopal Effect on a Distant Non-Irradiated Tumour.* Mol Pharm, 2022. **19**(11): p. 4199-4211.

9. Pei, P., et al., *Radionuclide labeled gold nanoclusters boost effective anti-tumour immunity for augmented radio-immunotherapy of cancer.* Nano Today, 2021. **38**: p. 101144.

10. Chao, Y., et al., *Combined local immunostimulatory radioisotope therapy and systemic immune checkpoint blockade imparts potent antitumour responses.* Nature Biomedical Engineering, 2018. **2**(8): p. 611-621.

11. Lekmeechai, S., K. Pietras, and O. Axelsson, *177Lu-SN201 nanoparticle shows superior anti-tumour efficacy over conventional cancer drugs in 4T1 orthotopic model.* Investigational New Drugs, 2024.

12. Institute of Medicine and National Research Council, *4, Targeted Radionuclide Therapy*, in *Advancing Nuclear Medicine Through Innovation*. 2007, The National Academies Press: Washington, DC. p. 173.

13. Huang, Y., et al., *64Cu/177Lu-DOTA-diZD, a Small-Molecule-Based Theranostic Pair for Triple-Negative Breast Cancer.* Journal of Medicinal Chemistry, 2021. **64**: p. 2705-2713.

14. Han, K. and I. Jung, *Restricted Mean Survival Time for Survival Analysis: A Quick Guide for Clinical Researchers.* Korean J Radiol, 2022. **23**(5): p. 495-499.

15. Patten, L.W., et al., *Assessing the performance of different outcomes for tumour growth studies with animal models.* Animal Models and Experimental Medicine, 2022. **5**(3): p. 248-257.
