# Supplementary figures and images for: Efficacy of combined targeted radionuclide therapy and immune checkpoint Inhibition in animal tumour models: a systematic review and meta-analysis of the literature
Source: Eur J Nucl Med Mol Imaging. 2025 Apr 26;52(12):4735–51. doi: 10.1007/s00259-025-07293-0 (PMC12491095; doi:10.1007/s00259-025-07293-0)

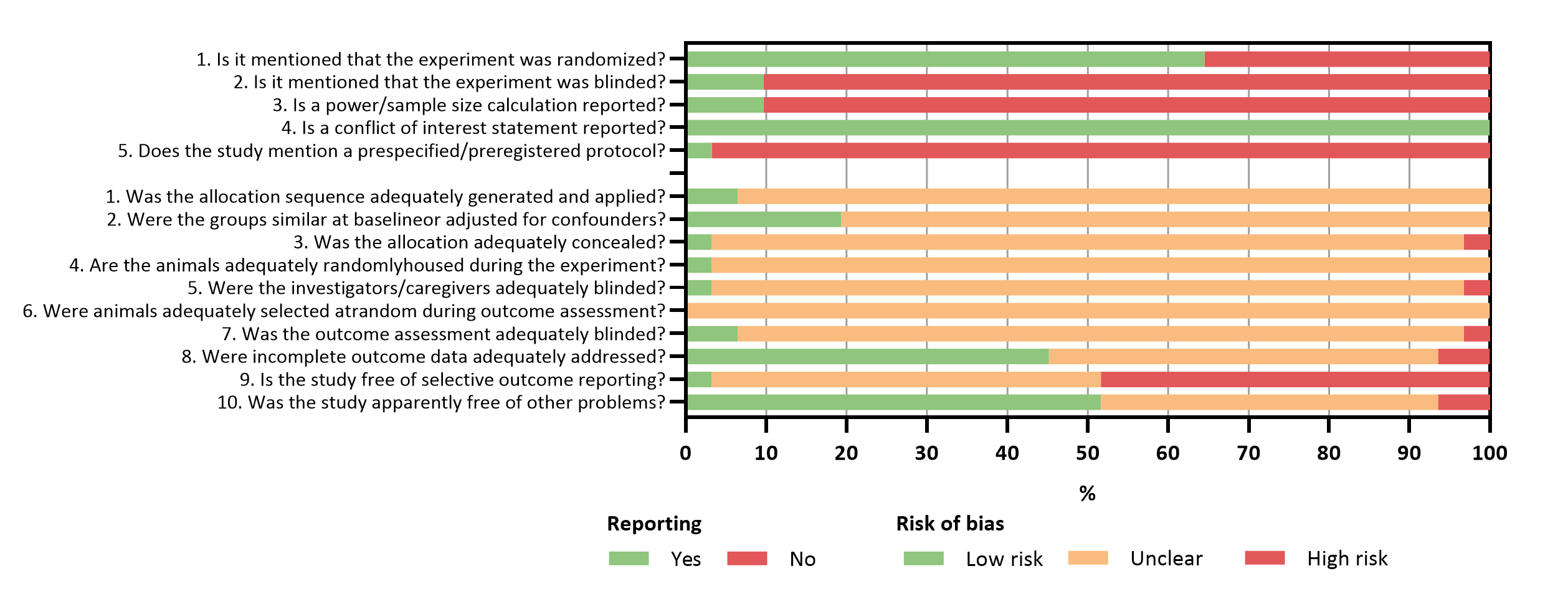

Supplement: Supplementary file 13 — Supplementary Material 13 [file 259_2025_7293_MOESM13_ESM.tif]
